# Association of Selenium Levels with Neurodegenerative Disease: A Systemic Review and Meta-Analysis

**DOI:** 10.3390/nu15173706

**Published:** 2023-08-24

**Authors:** Jiaxin Zhou, Wenfen Zhang, Zhiwen Cao, Shaoyan Lian, Jieying Li, Jiaying Nie, Ying Huang, Ke Zhao, Jiang He, Chaoqun Liu

**Affiliations:** 1 International School, Jinan University, Guangzhou 510080, China; zjx2001@stu2019.jnu.edu.cn; 2 School of Basic Medicine and Public Health, Jinan University, Guangzhou 510632, China; zwf1123@stu2021.jnu.edu.cn; 3 Center for Data Science, New York University, New York, NY 10011, USA; zc1592@nyu.edu; 4 Department of Nutrition, School of Medicine, Jinan University, Guangzhou 510632, China; lsy192735@163.com (S.L.); jieyingg77@163.com (J.L.); m18834183613@163.com (J.N.); hy19201920@163.com (Y.H.); zhaoke3612@163.com (K.Z.); 5 School of Biomedical Engineering, Southern Medical University, Guangzhou 510515, China; 6 Disease Control and Prevention Institute, Jinan University, Guangzhou 510632, China

**Keywords:** selenium, neurodegenerative disease, selenoprotein, systematic review, meta-analysis

## Abstract

Background: Neurodegenerative diseases (NDs) have posed significant challenges to public health, and it is crucial to understand their mechanisms in order to develop effective therapeutic strategies. Recent studies have highlighted the potential role of selenium in ND pathogenesis, as it plays a vital role in maintaining cellular homeostasis and preventing oxidative damage. However, a comprehensive analysis of the association between selenium and NDs is still lacking. Method: Five public databases, namely PubMed, Web of Science, EMBASE, Cochrane and Clinical Trials, were searched in our research. Random model effects were chosen, and Higgins inconsistency analyses (*I*^2^), Cochrane’s Q test and Tau2 were calculated to evaluate the heterogeneity. Result: The association of selenium in ND patients with Alzheimer’s disease (AD), Parkinson’s disease (PD), multiple sclerosis (MS), amyotrophic lateral sclerosis (ALS) and Huntington’s disease (HD) was studied. A statistically significant relationship was only found for AD patients (SMD = −0.41, 95% CI (−0.64, −0.17), *p* < 0.001), especially for erythrocytes. However, no significant relationship was observed in the analysis of the other four diseases. Conclusion: Generally, this meta-analysis indicated that AD patients are strongly associated with lower selenium concentrations compared with healthy people, which may provide a clinical reference in the future. However, more studies are urgently needed for further study and treatment of neurodegenerative diseases.

## 1. Introduction

Neurodegenerative diseases (NDs), such as Alzheimer’s disease (AD), Parkinson’s disease (PD), multiple sclerosis (MS), amyotrophic lateral sclerosis (ALS) and Huntington’s disease (HD) are debilitating and incurable disorders characterized by a progressive loss of neurons in the brain and spinal cord. The pathogenesis of NDs is multifaceted and diverse, yet they culminate in the common outcome of neuronal cell death. The global prevalence of NDs is on the rise, accompanied by diverse clinical manifestations, such as learning and cognition difficulty, memory impairment, memory distortion and sleeping disorders [1]. Unfortunately, due to the intricate and uncertain nature of their pathogenesis, there remains a profound lack of effective therapeutic modalities for these conditions.

It has been found that the altered homeostasis of several mineral elements is strongly associated with the progression of NDs; these elements include copper [2], iron [3,4] and selenium, a naturally nonmetallic element that widely exists in minerals, soil and food and is essentially required by the human body for normal physiological processes. It is considered as an irreplaceable element with key importance for keeping the body operational and healthy, for example by preventing cancers and cardiovascular diseases and regulating the immune system [5,6]. Significantly, it exhibits potent antioxidant properties, which are closely associated with NDs. Selenium mediates biological functions through its role in selenoproteins, acting in enhancing internal antioxidative defense mechanisms, protecting against oxidative injury [7,8]. It can also work as an antidote to toxicity of heavy metals and xenobiotics [9]. For instance, selenium compounds decrease the intracellular toxicity of deranged tau and Aβ and inhibit the accumulation of advanced glycation end-products and metal-induced neurotoxicity in AD [10]. Moreover, selenium can influence compounds with hormonal activity and neurotransmitters in the human brain, regulate calcium ion channels and modulate neurogenesis [11,12]. In addition, research has shown that increased selenium levels are associated with higher activity of glutathione peroxidase (GPx), an important antioxidant enzyme related to NDs [13]. These findings highlighted the potential clinical value of selenium in preventing and managing NDs.

Nowadays, the precise association and possible relationship between selenium and NDs still remain controversial. For example, Huseyin Vural et al. reported that the plasma in AD patients had lower levels of selenium compared with that in healthy individuals, and they established a strong relationship between selenium and GPx values [14]. However, some studies [15,16] have shown no statistically significant difference in selenium levels in AD patients. Interestingly, Sreeram Krishnan et al. even pointed that blood selenium is not involved in regulating oxidative stress in AD [17]. Some research suggested a protective mechanism of selenium in PD patients [18,19]. Contrarily, there are studies that have reported no significant relationship between the two variables [20]. The research focusing on selenium and MS is still insufficient. But it was reported that selenium supplementation was potentially related to MS improvement [21]. A study showed ALS patients presented higher SeO32 levels but lower organo-selenium compounds [22], but a higher ALS incidence was observed in people drinking water with a higher concentration of inorganic selenium [23]. As for HD patients, there is an obvious decrease in selenium content in specific regions of the brain, including the putamen, dorsolateral prefrontal cortex, primary visual cortex, cingulate gyrus and cerebellum [24]. But no difference was observed in the plasma of HD patients compared with healthy people.

NDs exhibit shared mechanisms at both the cellular and molecular levels, leading to the progressive degeneration of neurons. The convergence of these commonalities emphasizes the close interrelation and commonality among NDs. Currently, conflicting findings persist regarding the potential association between selenium levels and NDs. Furthermore, many studies about this relationship have been limited by small sample sizes and have not combined all five of these NDs. Therefore, the objective of this meta-analysis is to investigate the relationship between selenium levels and NDs, encompassing the conditions of AD, PD, MS, ALS and HD.

## 2. Methods and Materials

### 2.1. Protocol and Registration

The protocol for this research was registered in the International Prospective Register of Systematic Reviews (PROSPERO; CRD42021286946) and was carried out based on the Pattern of Reporting Systematic Review and Meta-Analysis (PRISMA) guideline.

### 2.2. Literature Searching Strategy

Five databases, namely PubMed, Web of Science, EMBASE, Cochrane Library and Clinical Trials, were retrieved using Mesh terms from National Center Biotechnology Information (NCBI), and the reference lists of eligible reports were thoroughly searched to identify potentially relevant literature. The databases were searched through titles and abstracts, using the keywords “selenium”, “Alzheimer’s disease”, “Parkinson’s disease”, “Multiple sclerosis”, “Amyotrophic lateral sclerosis” and “Huntington’s disease” and the Boolean operators “OR” and “AND”. Literature published between 1976 and 2023 July was searched. All of these studies were retrieved by two authors independently.

### 2.3. Inclusion and Exclusion Criteria

The criteria were formulated by all authors. Inclusion criteria encompassed the following aspects: (I) studies with the tests of selenium concentration in ND patients; (II) the research should contain both an ND case group and a healthy control group; (III) the studies should include standard and sufficient data; (IV) research data must be obtained independently by relative teams or organizations; (V) the publication language is English.

Exclusion criteria were as follows: (I) duplicate publications and data; (II) research data come from public databases; (III) literature types are reviews, case reports, meeting abstracts and basic experimental research literature; (IV) literature language is other than English.

### 2.4. Data Extraction and Quality Assessment

For each of the included studies, the following data were collected: author’s name, publication year, country or area, the number of patients, study design, ND type, tissue type, mean value of selenium concentration, SD (standard deviation) value. The information was reported in a standardized data extraction spreadsheet for further analysis. Furthermore, to assess the quality of the eligible studies, an independent evaluation was conducted using the Newcastle–Ottawa Scale. Additionally, funnel plots were utilized to assess potential bias and ensure the robustness of the findings.

### 2.5. Statistical Analysis

The meta-analysis was performed using the Review Manager (RevMan) software version 5.4, and the obtained results were considered statistically significant when *p* < 0.05. Random model effects were adapted to decrease the influence of heterogeneity, which was evaluated using Higgins inconsistency analyses (*I*^2^), Cochrane’s Q test, and Tau^2^.

## 3. Results

### 3.1. Study Selection

**Figure 1:**
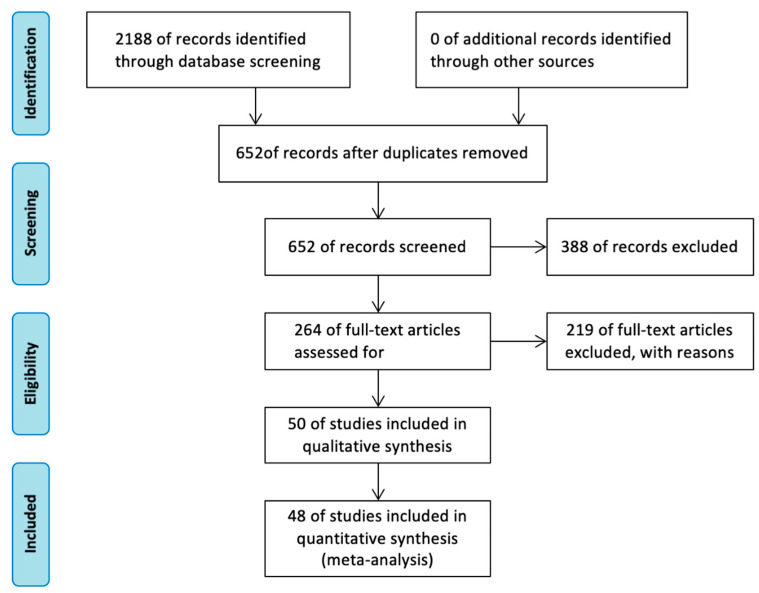
Flow diagram of the study search and selection in this meta-analysis.

The initial literature search identified 2188 reports in total, including 363 reports from PubMed, 562 reports from Web of Science, 1064 reports from EMBASE, 53 from Cochrane Library and 146 from Clinical Trials. After removing duplicate reports, 652 reports were considered to be potentially eligible. Upon careful evaluation based on the predefined inclusion and exclusion criteria, a final selection of 48 articles was made. The study selection flowchart is shown in Figure 1.

### 3.2. Baseline Characteristics of Included Studies

The eligible studies included 5334 participants and five ND types, 2377 individuals with AD, 1267 with PD, 659 with MS, 995 with ALS and 36 with HD. These studies were conducted in 18 different geographical areas and spanned publication years from 1976 to 2023. Among them, 48 studies had a case–control study design, while others had a nested case–control study design. The detailed information is presented in the tables below, Table 1 for ZD, Table 2 for PD, Table 3 for MS, Table 4 for ALS and Table 5 for HD.

### 3.3. Analysis of Selenium and AD

**Figure 2:**
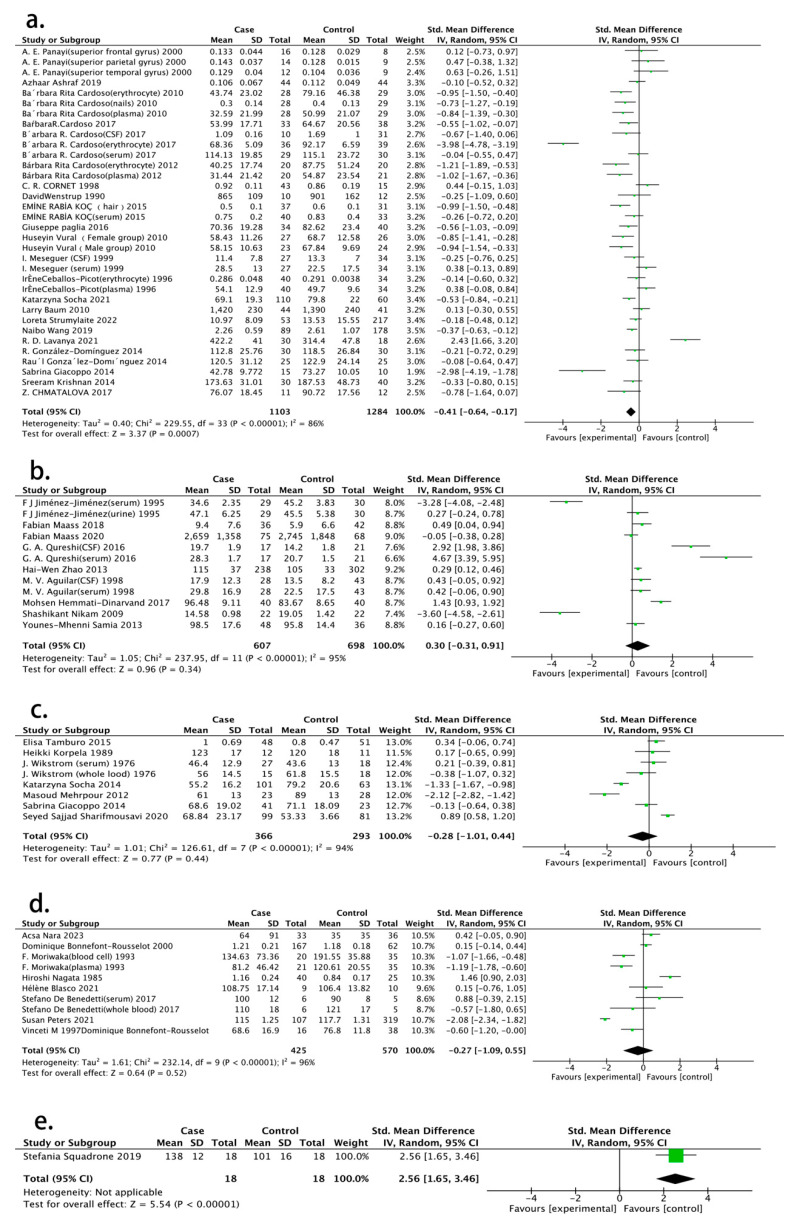
Forest plot showing the relationship between selenium level in neurodegenerative diseases patients and neurodegenerative diseases. (**a**) Relationship between selenium level in Alzheimer’s disease patients and Alzheimer’s disease [14,15,16,17,25,26,27,28,29,30,31,32,33,34,35,36,38,39,40,41,42,43]; (**b**) relationship between selenium level in Parkinson’s disease patients and Parkinson’s disease [44,45,46,47,48,49,50,51,52]; (**c**) relationship between selenium level in multiple sclerosis patients and multiple sclerosis [53,54,55,56,57,58,60,61,62,63,64,66]; (**d**) relationship between selenium level in amyotrophic lateral sclerosis patients and amyotrophic lateral sclerosis [59,60,61,62,63,64,66]; (**e**) relationship between selenium level in Huntington’s disease patients and Huntington’s disease [67]. Note: SD, standard deviation; Std. Mean Difference, standard mean difference; CI, confidence interval.

**Figure 3:**
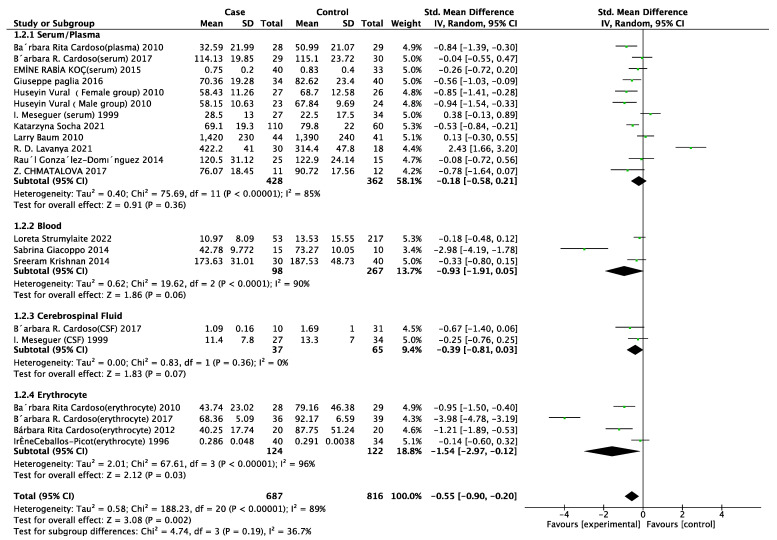
Subgroup analysis between selenium levels of different tissue types in Alzheimer’s disease patients and Alzheimer’s disease. Serum/Plasma subgroup [14,16,27,28,29,34,35,37,38,39,42]; Blood [17,31,43]; Cerebrospinal Fluid [16,34]; Erythrocyte [30,33,34,37]. Note: CSF, cerebrospinal fluid; SD, standard deviation; Std. Mean Difference, standard mean difference; CI, confidence interval.

In total, 48 studies were selected for the meta-analysis performed to observe the intrinsic association of selenium in AD patients. It was found that AD patients were associated with lower selenium concentrations in circulation compared with healthy people (SMD = −0.41, 95% CI (−0.64, −0.17), *p* < 0.001) (Figure 2a). Subgroup studies are necessary to obtain more details. Because selenium levels in patients were detected in different sample sources, we categorized them into four groups: serum/plasma, blood, cerebrospinal fluid (CSF) and erythrocytes. Only the erythrocyte group (SMD = −1.54, 95% CI (−2.97, −0.12), *p* = 0.03) displayed a substantial association with selenium in AD patients (Figure 3).

### 3.4. The Relationship between Selenium and PD, MS, ALS and HD

We included nine studies on PD, eight studies on MS and eight studies on ALS in our research for conducting the meta-analysis. However, no obvious statistical significance was found in selenium level between the patient group and the healthy control group, as shown in Figure 2b–d (PD: SMD =0.30, 95% CI (−0.31, 0.91), *p* = 0.34; MS: SMD = −0.28, 95% CI (−1.01, 0.44), *p* = 0.44; ALS: SMD = −0.27, 95% CI (−1.09, 0.55), *p* = 0.52). In the analysis of HD, only one study met the acceptance criteria and was identified (SMD = 2.56, 95% CI (1.65, 3.46)) (Figure 2e).

### 3.5. Assessment of Publication Bias

**Figure 4:**
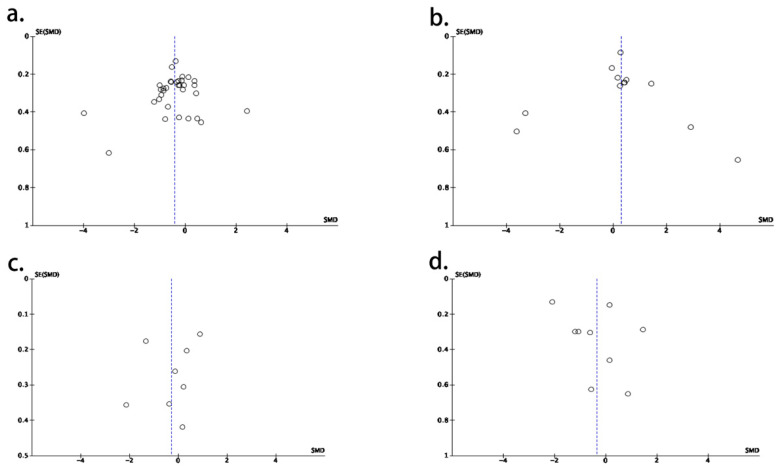
The funnel plots for the research for patients with different neurodegenerative diseases. (**a**) Analysis for Alzheimer’s disease; (**b**) analysis for Parkinson’s disease; (**c**) analysis for multiple sclerosis; (**d**) analysis for amyotrophic lateral sclerosis.

The publication bias for this research was evaluated by funnel plots, which are shown in Figure 4. There was no obvious publication bias found in this study. Newcastle–Ottawa Scale scores ranged from 5 to 9, as listed in Table 1. The heterogeneity value also indicated a low publication bias.

## 4. Discussion

Selenium, an essential element crucial for human health and development, plays a particularly vital role in preserving optimal brain function. In our research, it was demonstrated that AD patients are strongly associated with a lower selenium concentration in human, compared with healthy people, especially in erythrocyte. While no statistical significance was observed in PD, MS, ALS and HD patients.

The biological functions of selenium are mainly carried out by selenoprotein, including selenoprotein P (SelP), selenoprotein M (SelM), selenoprotein W (SelW), selenoprotein S (SelS), selenoprotein H (SelH). One of the mechanisms by which NDs develop is through deficiencies in antioxidant enzyme activity. The antioxidant activity of selenoprotein in CNS has been confirmed, related with neural diseases and decreased selenoprotein function. Selenium is an important element for the synthesis of selenoprotein, and also modulates their expression. Consequently, a decrease in selenium levels can result in compromised cognitive function and the onset of neurological disorders. Another hypothesis about NDs and selenium to consider is about reactive oxygen species (ROS) damage, which induces protein damage, misfolding, and aggregation, oxidative stress [68]. Glutathione peroxidases (GPxs), a selenoenzyme family capable of eliminating peroxides expressed in neurons and glia, can protect brain cells from ROS-induced damage. In animal experiments, the mice deficient in cytoplasmic GPxs showed increased infarct size and more apoptosis [69]. SleP has been reported to be able to deliver selenium into brain, protect brain from peroxynitrite-mediated oxidation and nitration, and combine with heavy metals as chelation antidote in brain [70,71,72,73,74,75]. It has been demonstrated as the most efficient selenium donor, with the ability to maintain selenium competence and GPx activity [76]. Inflammation is a complex biological response orchestrated by the immune system in response to harmful stimuli, which may also serve as one of the main factors to NDs. In the context of ND, chronic and persistent inflammation can contribute to the progression and exacerbation of neuronal damage. Activated immune cells, such as microglia and astrocytes, release pro-inflammatory molecules, which directly damage neurons and disrupt their normal functioning [77]. Furthermore, inflammatory processes can also disrupt the blood-brain barrier, which allows immune cells and inflammatory molecules to infiltrate the brain, further fueling the inflammatory response [78]. Previous studies have provided evidences suggesting that selenium possesses the ability to attenuate inflammatory mediators through the inhibition of nuclear factor kappa-B (NF-kB) expression [79]. Given its antioxidant and anti-inflammatory properties, selenium appears to possess the capability to effectively enhance glucose metabolism by mitigating inflammation.

### 4.1. AD

Progressive loss of memory and impairment of daily activities have become the main characteristics of AD patients. Oxidative stress is a significant part of AD pathogenesis, and ROS can adversely affect the mitochondrial biofunction, synaptic transmission, axonal transport, and stimulate neuroinflammation [80,81,82]. In this case, GPx has performed the central defensive role in AD [37]. Selenium provides the protection to cellular tissue from ROS-induced cell damage with the proposed mechanisms invoking the functions of GPxs and SleP [83]. In our study, we observed a significant reduction in selenium levels among patients with AD (*p* < 0.001), which may indicate a worse antioxidant ability and stronger ROS attack in AD patients because of the lower selenium level. In addition, we observed variations in the significance of selenium concentration analysis across different tissues, particularly significant in erythrocyte level, while no statistical difference was found in serum/plasma, blood and CSF. This result arises us regarding the reason for the opposite significance finding. And furthermore, it interests us to explore the relationship between selenium content in different tissue regions and their discrepancy in neurological functional implications. As the plasma is considered as a marker of recent exposure, while erythrocytes tend to reflect longer-term selenium level, which may suggest that the infection of selenium in AD patients is a gradual and long-term procedure, rather than rapid alteration. In that case, only in erythrocyte can we observe a loss of selenium. And there may exist some compensatory ways which can maintain the selenium balance in plasma. Human brain is the most metabolically active organ in the body, with only 2% total mass but about 20% of the whole oxygen need and 25% glucose consumption of whole body [84]. In addition, selenoproteins regulate glucose metabolism and the expression of genes involved in glycolysis and gluconeogenesis [85]. Thus, selenium is strongly associated with the brain organ, the organ reliant on glycolysis as its primary energy source. Compared with other organs, the selenium concentration in brain is low. However, it has the highest priority for the uptake and retention of selenium when deficiency happens [86]. According to this mechanism, the CSF selenium amount in AD patients may be kept in a normal range, which can reasonably match with our finding.

### 4.2. PD

PD is the ND resulting primarily from the loss of dopaminergic neurons in substantia nigra, characterized with the presence of intraneuronal proteinaceous inclusions termed Lewy bodies. Current medications only treat on symptoms of PD rather than stopping the neuron degeneration. It was observed that the density of GPx-immunostained cells around the surviving dopaminergic neurons increased in PD patients, directly proportional to the extent of the severity in dopaminergic cell loss in the respective cell groups [87]. The research has found that, in patients with PD, the enzyme activity of GPx decreased about 20% in substantia nigra, external globus pallidus, putamen, and frontal cortex, compared with healthy people [88]. The value of selenium is strongly associated with GPx, which may suggest potential interactions [89]. While it is surprisingly that, in our research, after integrating the data, no statistically significant relationship was found between selenium and PD (*p* = 0.25), neither in serum nor CSF, similar with another study [90]. This finding may be attributed to the presence of a robust self-regulating mechanism within the human body during the pathogenesis of PD.

### 4.3. MS

MS is a kind of progressive, inflammatory chronic disease affecting the function of CNS. The etiology of MS remains unclear, while it is believed to be associated with genetic and environmental interaction, abnormal immune response, oxidative stress [91]. Currently, there is no evidence or research showing a direct relationship between selenium and MS. Meanwhile, no significant relationship was observed between selenium and MS in our study (*p* = 0.45), which may suggest multiple effect factors between selenium and MS, such as immunology, gene and etc., requiring more research.

### 4.4. ALS

ALS, a devastating motor neuron disease of unknown etiology, has been linked to genetic factors in certain cases, although the precise mechanisms remain to be fully elucidated. Vinceti et al. had described that the elevated level of selenite and selenised human serum albumin in CSF is related with higher sporadic ALS risk, while an elevated CSF SePP level was found to be linked with lower risk of this disease [62]. However, this point remains controversial. Some other studies showed that reduced selenium level was observed in ALS patients compared with healthy people [64]. Nagata H et al. and his team reported a higher selenium level in the red blood cells of patients with ALS [65]. Moreover, several related studies have reported no significant difference of the selenium levels in body fluids was observed between ALS patients and healthy people [59,60]. Thus, the association of selenium levels and ALS was investigated in our research. We found that ALS patients had a tendency toward decreased total selenium levels compared with healthy, but without statistically significant difference (*p* = 0.64). Hence, further investigations are necessary to address the association of selenium levels in ALS individuals.

### 4.5. HD

HD, a dominantly inherited neurodegenerative disorder that results from the expansion of polyglutamine repeat in the huntingtin (HTT) gene [92]. In the population of HD, it was found that not only selenium inhibits lipid peroxidation by increasing specific glutathione peroxidases, but also GPx activity in the brain was significantly increased, especially GPx1 and GPx6 [93]. Increased ROS production may result in a stress situation that activates several pathways to increase the expression of antioxidant enzymes [94]. However, in the included study of our research, HD patients got a higher selenium level compared with healthy people [67]. Oxidative stress and genetics seem to be a key process to understand the pathogenesis of HD, but the intrinsic mechanism is still unclear. And more detailed research was still lacked.

This research has been the most comprehensive analysis involving all of the five NDs with the largest number of studies and participants, which offers potential clinical value and can drive further research in the field of treatment strategies. Nevertheless, some limitations still exist in the current research. Firstly, the heterogeneity of included studies cannot be ignored. Various region and race, large span of publication year, the interaction between selenium and other food or drugs can result in high heterogeneity. In addition, although we recorded the baseline characteristics of the population and did the subgroup analysis as comprehensive as possible, more detailed investigations are still warranted to explore the intricate relationships and provide valuable insights into the potential therapeutic implications of selenium in this NDs.

## 5. Conclusions

In conclusion, our research revealed that AD patients are strongly associated with lower selenium concentration compared with healthy people, particularly in the erythrocyte, providing a meaningful reference to clinic. While there is no statistically significant difference between selenium level and PD, MS, ALS and HD. Still, more studies are needed to find the concrete mechanism between selenium level and NDs and further improve the clinical treatment.

## Figures and Tables

**Table 1:** Basic characteristics of the studies on Alzheimer’s disease.

Study	Year	Country	Tissue Type	Patients		Healthy Controls		Unit	Study Type	NOS Score
				No.	Selenium Level (Mean ± Sd.)	No.	Selenium Level (Mean ± Sd.)			
Huseyin Vural [14]	2010	Turkey	Plasma	23M	58.15 ± 10.63	24M	67.84 ± 9.69	μg/L	Case–control	8
				27F	58.43 ± 11.26	26F	68.70 ± 12.58			
Naibo Wang [25]	2019	China	Urine and Blood	89	2.26 ± 0.59	178	2.61 ± 1.07	umol/mL	Nested case–control	6
I. Meseguer [16]	1999	Spain	CSF	27	11.40 ± 7.80	34	13.30 ± 7.00	ng/mL	Case–control	5
			Serum	27	28.50 ± 13.00	34	22.50 ± 17.50			
R.González-Domínguez [26]	2014	Spain	Serum	30	112.80 ± 25.76	30	118.50 ± 26.84	μg/L	Case–control	8
EMİNE RABİA KOÇ [27]	2015	Turkey	Hair	37	0.50 ± 0.10	31	0.60 ± 0.10	µg/g	Case–control	7
			Serum	40	0.75 ± 0.20	33	0.83 ± 0.40	µg/mL		
Katarzyna Socha [28]	2021	Poland	Serum	110	69.10 ± 19.30	60	79.80 ± 22.00	μg/L	Case–control	7
Giuseppe paglia [29]	2016	Italy	Serum	34	70.36 ± 19.28	40	82.62 ± 23.40	μg/L	Case–control	6
Sreeram Krishnan [17]	2014	India	Blood	30	173.63 ± 31.01	40	187.53 ± 48.73	ppb	Case–control	8
Bárbara Rita Cardoso [30]	2012	Brazil	Plasma	20	31.44 ± 21.42	21	54.87 ± 23.54	μg/L	Case–control	7
			Erythrocytes	20	40.25 ± 17.74	20	87.75 ± 51.24			
Sabrina Giacoppo [31]	2014	Italy	Blood	15	42.78 ± 9.772	10	73.27 ± 10.05	μg/L	Case–control	5
Azhaar Ashraf [32]	2019	NK	plasma	44	0.106 ± 0.067	44	0.112 ± 0.049	μg/L	Case–control	6
IrèneCeballos-Picot [33]	1996	France	Plasma	40	54.10 ± 12.90	34	49.70 ± 9.60	ng/mL	Case–control	6
			Erythrocytes	40	0.286 ± 0.048	34	0.291 ± 0.0038			
Bárbara R. Cardoso [34]	2017	Australia	Erythrocytes	36	68.36 ± 5.09	39	92.17 ± 6.59	μg/L	Case–control	6
			Serum	29	114.13 ± 19.85	30	115.10 ± 23.72			
			CSF	10	1.09 ± 0.16	31	1.69 ± 1.00			
Larry Baum [35]	2010	China	Serum	44	1420 ± 230	41	1390 ± 240	nmol/L	Case–control	5
David Wenstrup [36]	1990	USA	Whole tissue	10	865 ± 109	12	901 ± 162	ng/g	Case–control	5
C. R. CORNET [15]	1998	USA	Pituitary tissues	43	0.92 ± 0.11	15	0.86 ± 0.19	μg/g	Case–control	5
Bárbara Rita Cardoso [37]	2010	Brazil	Plasma	28	32.59 ± 21.99	29	50.99 ± 21.07	μg/g	Case–control	7
			Erythrocytes	28	43.74 ± 23.02	29	79.16 ± 46.38			
			Nails	28	0.3 ± 0.14	29	0.4 ± 0.13			
Z. CHMATALOVA [38]	2017	Czech Republic	Plasma	11	76.07 ± 18.45	12	90.72 ± 17.56	μg/L	Case–control	6
Raúl González-Domínguez [39]	2014	NK	Serum	25	120.5 ± 31.12	15	122.9 ± 24.14	μg/L	Case–control	5
A. E. Panayi [40]	2000	Netherlands	Superior frontal gyrus (dry mass)	16	0.133 ± 0.044	8	0.128 ± 0.029	μg/L	Case–control	6
			Superior parietal gyrus (dry mass)	14	0.143 ± 0.037	9	0.128 ± 0.015			
			Superior temporal gyrus (dry mass)	12	0.129 ± 0.040	9	0.104 ± 0.036			
BáŕbaraR.Cardoso [41]	2017	America	Membrane fraction of wet tissue	33	53.99 ± 17.71	38	64.67 ± 20.56	ng/g	Case–control	5
R. D. Lavanya [42]	2021	India	Serum	30	422.2 ± 41.0	18	314.4 ± 47.8	ng/g	Case–control	5
Loreta Strumylaite [43]	2022	Italy	NK	53	10.97 ± 8.09	217	13.53 ± 15.55	kg × 10^−9^/m^3^ × 10^−3^	Case–control	6

Abbreviations: M, male; F, female; NOS score, Newcastle–Ottawa Scale score; NK, not known; No., number of participants; Sd, standard deviation.

**Table 2:** Basic characteristics of the studies on Parkinson’s disease.

Study	Year	Country	Tissue Type	Patients		Healthy Controls		Unit	Study Type	NOS Score
				No.	Selenium Level (Mean ± Sd.)	No.	Selenium Level (Mean ± Sd.)			
Fabian Maass [44]	2020	Germany	CSF	75	2695 ± 1358	68	2745 ± 1848	ng/L	Case–control	6
Mohsen Hemmati-Dinarvand [45]	2017	Iran	Serum	40	96.48 ± 9.11	40	83.67 ± 8.65	μg/L	Case–control	5
Hai-Wen Zhao [46]	2013	China	Plasma	238	115 ± 37	302	105 ± 33	μg/L	Case–control	7
M. V. Aguilar [47]	1998	Spain	CSF	28	17.9 ± 12.3	43	13.5 ± 8.2	ng/mL	Case–control	7
			Serum	28	29.8 ± 16.9	43	22.5 ± 17.5			
F J Jiménez-Jiménez [48]	1995	Spain	Serum	29	34.60 ± 2.35	30	45.20 ± 3.83	μg/L	Case–control	6
			Urine	29	47.10 ± 6.25	30	45.50 ± 5.38	μg/24 h		
Fabian Maass [49]	2018	Germany	CSF	36	9.4 ± 7.6	42	5.9 ± 6.6	μg/L	Case–control	6
Shashikant Nikam [50]	2009	India	Plasma	22	14.58 ± 0.98	22	19.05 ± 1.42	μgm/dL	Case–control	7
Younes-Mhenni Samia [51]	2013	Tunisia	Plasma	48	98.5 ± 17.6	36	95.8 ± 14.4	μg/L	Case–control	6
G. A. Qureshi [52]	2016	Sweden	CSF	17	19.7 ± 1.9	21	14.2 ± 1.8	μg/L	Case–control	5

Abbreviations: NOS score, Newcastle–Ottawa Scale score; No., number of participants; Sd, standard deviation.

**Table 3:** Basic characteristics of the studies on multiple sclerosis.

Study	Year	Country	Tissue Type	Patients		Healthy Controls		Unit	Study Type	NOS Score
				No.	Selenium Level (Mean ± Sd.)	No.	Selenium Level (Mean ± Sd.)			
Seyed Sajjad Sharifmousavi [53]	2020	Iran	Plasma	99	68.84 ± 23.17	81	53.33 ± 3.66	μg/L	Case–control	7
Katarzyna Socha [54]	2014	Poland	Serum	101	55.2 ± 16.2	63	79.2 ± 20.6	μg/L	Case–control	6
J. Wikstrom [55]	1976	Finland	Whole blood	15	56.0 ± 14.5	18	61.8 ± 15.5	ng/mL	Case–control	5
			Serum	27	46.4 ± 12.9	18	43.6 ± 13.0			
Elisa Tamburo [56]	2015	Italy	Hair	48	1.00 ± 0.69	51	0.8 ± 0.47	μg/g	Case–control	7
Masoud Mehrpour [57]	2012	Iran	Serum	23	61 ± 13	28	89 ± 13	μg/L	Case–control	5
Sabrina Giacoppo [31]	2014	Italy	Blood	41	68.60 ± 19.02	23	71.10 ± 18.09	μg/L	Case–control	5
Heikki Korpela [58]	1989	Finland	Serum	12	123 ± 17	11	120 ± 18	μg/L	Nested Case–control	5

Abbreviations: NOS score, Newcastle–Ottawa Scale score; No., number of participants; Sd, standard deviation.

**Table 4:** Basic characteristics of the studies on amyotrophic lateral sclerosis.

Study	Year	Country	Tissue Type	Patients		Healthy Controls		Unit	Study Type	NOS Score
				No.	Selenium Level (Mean ± Sd.)	No.	Selenium Level (Mean ± Sd.)			
Stefano De Benedetti [59]	2017	Italy	Serum	6	100 ± 12	5	90 ± 8	μg/L	Case–control	7
			whole blood	6	110 ± 18	5	121 ± 17			
Vinceti M [60]	1997	Italy	Serum	16	68.6 ± 16.9	38	76.8 ± 11.8	μg/L	Case–control	7
Dominique Bonnefont-Rousselot [61]	2000	France	Plasma	167	1.21 ± 0.21	62	1.18 ± 0.18	μmol/L	Case–control	6
Susan Peters [62]	2021	NK	Erythrocytes	107	115 ± 1.25	319	117.70 ± 1.31	ng/g	Nested Case–control	6
Hélène Blasco [63]	2021	France	Blood	9	108.75 ± 17.14	10	106.40 ± 13.82	μg/L	Case–control	6
F. Moriwaka [64]	1993	Japan	Plasma	21	81.20 ± 46.42	35	120.61 ± 20.55	ng/g	Case–control	6
			Blood cells	20	134.63 ± 73.36	35	191.55 ± 35.88			
Hiroshi Nagata [65]	1985	Japan	Blood cells	40	1.16 ± 0.24	25	0.84 ± 0.17	ng/mg	Case–control	5
Acsa Nara [66]	2023	Brazil	Plasma	33	64.00 ± 91.00	36	35 ± 35	μg/L	Case–control	5

Abbreviations: NOS score, Newcastle–Ottawa Scale score; No., number of participants; Sd, standard deviation.

**Table 5:** Basic characteristics of the studies on Huntington’s disease.

Study	Year	Country	Tissue Type	Patients		Healthy Controls		Unit	Study Type	NOS Score
				No.	Selenium Level (Mean ± Sd.)	No.	Selenium Level (Mean ± Sd.)			
Stefania Squadrone [67]	2019	Italy	Blood	18	138 ± 12	18	101 ± 16	μg/L	Case–control	5

Abbreviations: NOS score, Newcastle–Ottawa Scale score; No., number of participants; Sd, standard deviation.

## Data Availability

The data and materials used to support the findings in this research are available from the corresponding author upon request.
